# Intravascular Ultrasound-Guided Iliofemoral Venous Stenting: In-Stent Mural Thrombus as a Possible Early Sign of Occult Malignancy

**DOI:** 10.7759/cureus.116395

**Published:** 2026-09-16

**Authors:** Yuki Tada, Shinsuke Kikuchi, Takayuki Uramoto, Naoya Kuriyama, Nobuyoshi Azuma

**Affiliations:** 1 Department of Vascular Surgery, Asahikawa Medical University, Asahikawa, JPN

**Keywords:** antithrombotic therapy, chronic venous obstruction, duplex ultrasound surveillance, iliofemoral vein, in-stent thrombus, intravascular ultrasound, malignancy risk, post-thrombotic syndrome, venous stent

## Abstract

Dedicated venous stents became available in Japan only recently, and reports of their use and follow-up in Japanese practice remain scarce. A 77-year-old woman developed acute left iliofemoral deep vein thrombosis (DVT) after prolonged recumbency and was treated with edoxaban. Eighteen days later she underwent endovascular therapy of the right lower limb for chronic limb-threatening ischaemia, and aspirin was added. Limb swelling and pain persisted despite three months of anticoagulation and graduated compression, and imaging then showed chronic occlusion of the left common and external iliac veins (EIV) with patent femoral and deep femoral inflow, corresponding to a favourable anatomical category of chronic venous obstruction (CVO). Under intravascular ultrasound (IVUS) guidance, which identified a stenotic distal landing zone in the proximal common femoral vein (CFV) that was not evident on venography, two Venovo venous stents (Becton, Dickinson and Company (BD), Tempe, AZ, USA) were deployed from the common iliac vein (CIV) to the CFV. Symptoms and clinical severity scores improved over the following months. Three months after stenting, duplex surveillance detected a localised mural thrombus along the posterior wall of the stent despite treatment with edoxaban and aspirin; the antiplatelet agent was changed to clopidogrel, yet the thrombus enlarged from 3 to 6 mm over the next six months before reaching a plateau. Nine months after stenting, an occult breast carcinoma was diagnosed incidentally. The stent remained patent at 12 months, through two bleeding events and three interruptions of antithrombotic therapy. This case illustrates one approach to managing an asymptomatic in-stent mural thrombus and raises the possibility that a thrombus forming despite adequate antithrombotic therapy may be an early manifestation of an undiagnosed malignancy.

## Introduction

Endovascular stenting is an established treatment for symptomatic chronic venous obstruction (CVO) of the iliofemoral segment [1]. In Japan, its use is governed by appropriate use criteria issued jointly by five professional societies, which restrict treatment to post-thrombotic disease of at least three months' duration with constant pain that markedly impairs daily life, adequate deep venous inflow into the common femoral vein, prior compression therapy, and imaging evidence of iliac occlusion or haemodynamically significant stenosis [2].

The Venovo venous stent system (Becton, Dickinson and Company (BD), Tempe, AZ, USA) was the first stent designed specifically for the iliofemoral segment to reach the Japanese market. It became reimbursable on 1 December 2024, and its use is restricted to certified institutions under a mandatory all-case post-marketing surveillance programme [2]. Published Japanese experience with its use and serial follow-up therefore remains limited, and the present case was treated within the first year of clinical availability.

The anatomical extent of obstruction is a key determinant of stent patency. A recently proposed anatomical classification of CVO of the iliofemoral tract distinguishes categories by how far the obstruction extends distally: type 3 denotes obstruction of the iliac veins extending to the common femoral vein (CFV) but sparing the deep inflow veins, whereas types 4 and 5 involve the femoral or deep femoral veins. Both the extent of obstruction and the number of stents were independently associated with loss of primary patency, and disease involving the deep inflow veins predicted poorer outcomes [3].

We report an early Japanese case of Venovo stenting for a post-thrombotic iliac occlusion, in which serial duplex surveillance detected an in-stent mural thrombus that arose despite combined anticoagulant and antiplatelet therapy, and in which an occult malignancy was diagnosed six months later.

## Case presentation

A 77-year-old woman presented with acute swelling and pain of the left lower limb. She weighed 60 kg, and her estimated glomerular filtration rate was 96.0 mL/min/1.73 m². Computed tomography (CT) at onset demonstrated acute deep vein thrombosis (DVT) of the left iliofemoral system (Figure 1A). The thrombosis was considered provoked by prolonged recumbency during an episode of bronchitis, and thrombophilia testing was therefore not undertaken. There were no clear anatomical features of May-Thurner compression. Anticoagulation with edoxaban 60 mg once daily was started, together with graduated compression stockings providing 20 mmHg.

**Figure 1:**
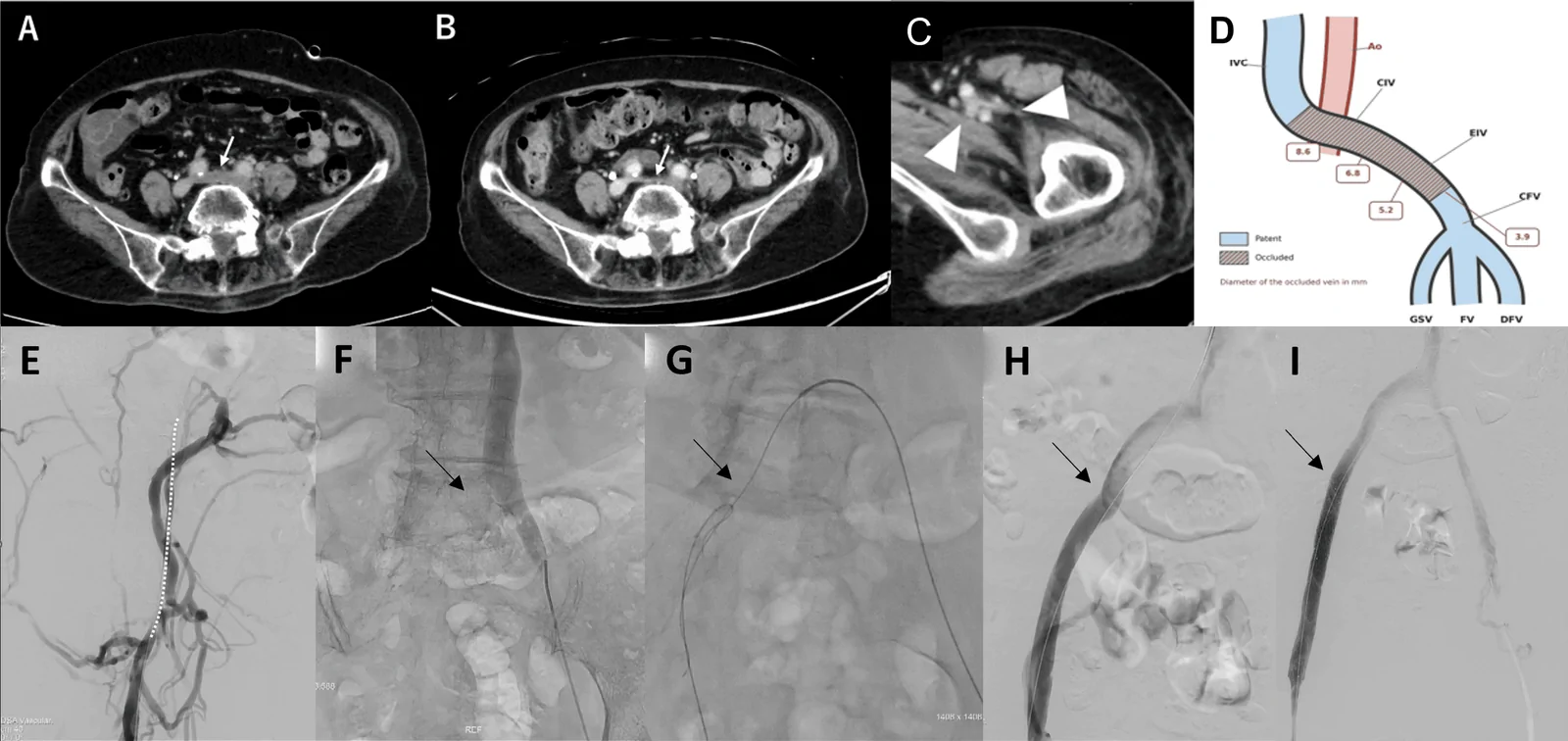
Diagnostic imaging, anatomical classification, and endovascular recanalization of a chronic post-thrombotic iliofemoral occlusion Diagnostic imaging: (A) Axial contrast-enhanced computed tomography (CT) at onset; the arrow indicates acute thrombus filling the left iliofemoral vein. (B) Axial contrast-enhanced CT at three months; the arrow indicates the persistently occluded left iliac vein. (C) Axial contrast-enhanced CT; the arrowheads indicate the patent FV and DFV. Anatomical classification: (D) Schematic venous map derived from duplex ultrasound; blue shading marks patent segments and hatching marks the occluded segment, which extends from the common iliac vein to the distal EIV and ends proximal to the CFV. The red numerals give the diameter of the occluded vein in millimeters, and the key is inset at the lower left. Image credits: Shinsuke Kikuchi. Panel D was prepared using Microsoft PowerPoint (Microsoft Corporation, Redmond, WA, USA). Endovascular recanalization: (E) Prone venogram of the left limb; the dotted line traces the occluded iliac vein, with a well-developed internal iliac collateral pathway. (F) Contralateral venogram; the arrow indicates the origin of the left CIV. (G) The arrow indicates the point at which the guidewire crossed the occlusion. (H) Venogram after dilation with an Atlas Gold 14 x 60-mm balloon (Becton, Dickinson and Company (BD), Tempe, AZ, USA); the arrow indicates the recanalized iliac vein. (I) Completion venogram; the arrow indicates the left iliac vein after deployment of two Venovo venous stents (BD, Tempe, AZ, USA). Abbreviations: Ao, aorta; CFV, common femoral vein; CIV, common iliac vein; DFV, deep femoral vein; EIV, external iliac vein; FV, femoral vein; GSV, great saphenous vein; IVC, inferior vena cava.

Eighteen days later she underwent endovascular therapy of the right lower limb for chronic limb-threatening ischaemia (WIfI stage W2 I1 fI0; ankle-brachial index 0.75 on the right and 0.78 on the untreated left, with significant stenoses at the origin of the superficial femoral artery and the above-knee popliteal artery). Aspirin was started for this arterial indication, and the edoxaban dose was reduced to 30 mg once daily, consistent with the Japanese label for patients weighing 60 kg or less and with the additional bleeding risk of concurrent antiplatelet therapy. She remained on edoxaban 30 mg thereafter.

Limb swelling and pain persisted despite three months of anticoagulation and compression. At that time, she had a symptomatic post-thrombotic limb: CEAP clinical class C3 [4], revised Venous Clinical Severity Score (rVCSS) 8 with a pain subscore of 3 [5], Villalta score 9 [6], numeric pain rating scale (NPRS) 3, and Venous Disability Score (VDS) 2 [5], with a calf circumference of 31.5 cm in the affected limb and 29.0 cm on the contralateral side. All scores were obtained at the same pre-intervention visit. Calf circumference was measured 20 cm distal to the knee joint line. Contrast-enhanced CT confirmed persistent total occlusion of the left iliac vein with developed collateral pathways (Figure 1B) and patent femoral and deep femoral inflow (Figure 1C).

Duplex ultrasound (DUS) showed no flow in the left common and external iliac veins (EIV), which were occluded and reduced in calibre, while the great saphenous vein (GSV), femoral vein (FV), and deep femoral vein (DFV) were patent with antegrade inflow and without significant deep or superficial venous reflux (Figure 1D). These features were consistent with an organised post-thrombotic obstruction rather than an acutely distended thrombosed vein. Because the obstruction involved the iliac and iliofemoral segment above the confluence of the CFV while both deep inflow veins remained patent, the lesion corresponded to type 3 of the proposed CVO classification, a category previously associated with favourable patency [3]. The patient, therefore, met the national appropriate use criteria for venous stenting: post-thrombotic disease of more than three months' duration, CEAP clinical class C3 with an rVCSS pain subscore of 3, persistent symptoms despite anticoagulation and compression therapy, preserved deep venous inflow, and imaging evidence of iliac occlusion with collateral development [2]. Venous intervention was, therefore, indicated.

The procedure was performed under general anaesthesia in the prone position. The affected left popliteal vein was punctured under ultrasound guidance, and a 5-Fr 11-cm sheath was placed. Venography confirmed chronic total occlusion of the left external and common iliac veins (CIVs, Figure 1E). The sheath was then exchanged for a 9-Fr 25-cm sheath (Terumo Corporation, Tokyo, Japan). Antegrade crossing from the ipsilateral access proved difficult, and the contralateral right popliteal vein was therefore accessed with an identical 5-Fr sheath for venography and to establish a pull-through wire. Right-sided venography delineated the origin of the left CIV and the inferior vena cava (Figure 1F), which also allowed us to confirm that the proximal stent end would not protrude into the cava. The left CIV was crossed retrogradely using a 4-Fr 110-cm Tempo multipurpose catheter (Cordis, Miami Lakes, FL, USA), and the chronic total occlusion was crossed using a pull-through technique (Figure 1G). All stents were subsequently delivered through the ipsilateral popliteal access.

Pre-dilatation with an 8.0 x 100-mm Mustang balloon (Boston Scientific, Marlborough, MA, USA) at rated pressure restored antegrade flow, and the segment from the CIV to the proximal CFV was then dilated with an Atlas Gold 14 x 60-mm balloon (BD, Tempe, AZ, USA) at nominal pressure (Figure 1H). After confirming the inferior vena cava confluence by contralateral venography, a Venovo 14 x 140-mm stent was deployed in the iliac segment, selected according to the manufacturer's sizing table, which specifies a stent 1 to 3 mm larger than the reference vessel diameter. To extend coverage into the CFV, a Venovo 12 x 140-mm stent was placed at the proximal CFV across the residual lesion; a smaller diameter was chosen for this segment because an oversized stent risks impingement on the femoral confluence. The two stents overlapped by 32 mm. Post-dilatation was performed with Atlas Gold balloons matched to each stent, 14 x 60 mm for the iliac segment and 12 x 60 mm for the segment across the CFV. Completion venography showed good in-line flow (Figure 1I).

Intravascular ultrasound (IVUS; Visions PV .035, Philips, San Diego, CA, USA) was used at each stage of the procedure (Figure 2). Before dilatation, it delineated the true lumen and the chronic circumferential wall thickening of the occluded segment. The minimum lumen area was 35.6 mm² in the CIV, 49.4 mm² in the EIV, and 55.6 mm² in the proximal CFV. The common iliac lumen was markedly flattened, with maximum and minimum diameters of 11.1 and 4.6 mm, respectively, giving a ratio of 2.4. The proximal CFV was stenotic, a finding not apparent on venography, and this prompted extension of stent coverage into that segment. True-lumen measurements guided balloon and stent sizing and defined the proximal and distal landing zones. After balloon dilatation, the corresponding areas were 65.0 mm², 114.1 mm², and 112.6 mm², and after stenting 149.9 mm², 160.6 mm², and 122.6 mm², giving equivalent diameters of 13.8 mm, 14.3 mm, and 12.5 mm, consistent with the nominal 14 mm and 12 mm stent diameters. IVUS also confirmed adequate expansion and wall apposition at every level.

**Figure 2:**
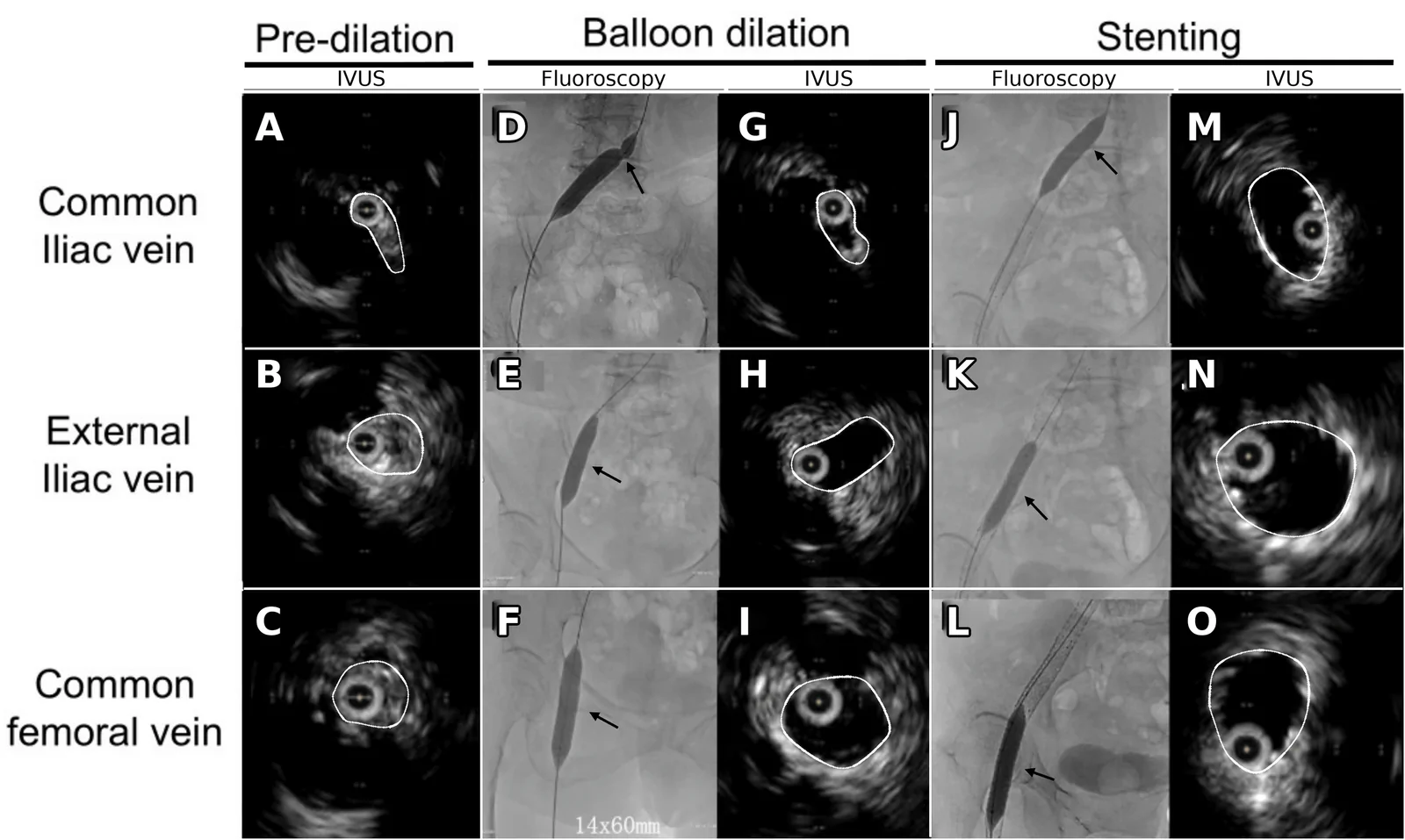
Intravascular ultrasound and fluoroscopic findings at each stage of endovascular recanalization Each row corresponds to a venous level, from the common iliac vein at the top to the common femoral vein at the bottom. The five columns follow the procedural sequence from left to right, and panels are lettered from top to bottom within each column. The dotted line outlines the venous lumen on every intravascular ultrasound panel. Pre-dilation: (A) Common iliac vein; (B) External iliac vein; (C) Common femoral vein. The post-thrombotic lumen is narrowed and surrounded by chronic circumferential wall thickening at all three levels. The common iliac lumen is markedly flattened, and the proximal common femoral vein is stenotic, a finding that was not apparent on venography and that prompted extension of stent coverage into that segment. Balloon dilation: (D, E, F) Fluoroscopy during dilation with an Atlas Gold 14 x 60-mm balloon (Becton, Dickinson and Company (BD), Tempe, AZ, USA); the arrow in (D) indicates the residual waist at the stenosis, and the arrows in (E) and (F) indicate the dilated segment. (G, H, I) Intravascular ultrasound after balloon dilation, showing the restored lumen. Stenting: (J, K, L) Fluoroscopy during post-dilatation of the deployed Venovo venous stents (BD, Tempe, AZ, USA); the arrows indicate the balloon inflated within the stent. (M, N, O) Intravascular ultrasound after stenting, showing a fully expanded and well-apposed stent at each level.

Intraprocedural heparin was administered to maintain an activated clotting time above 250 seconds. Heparin 10,000 units per day was continued until the following morning in addition to edoxaban, because the patient was confined to bed on the day of the procedure; edoxaban was not interrupted. Plain radiography after the procedure confirmed satisfactory stent position and expansion (Figure 3A), and DUS on postoperative day one confirmed a patent stent (Figure 3B). She continued edoxaban 30 mg and aspirin, and compression stockings were maintained.

**Figure 3:**
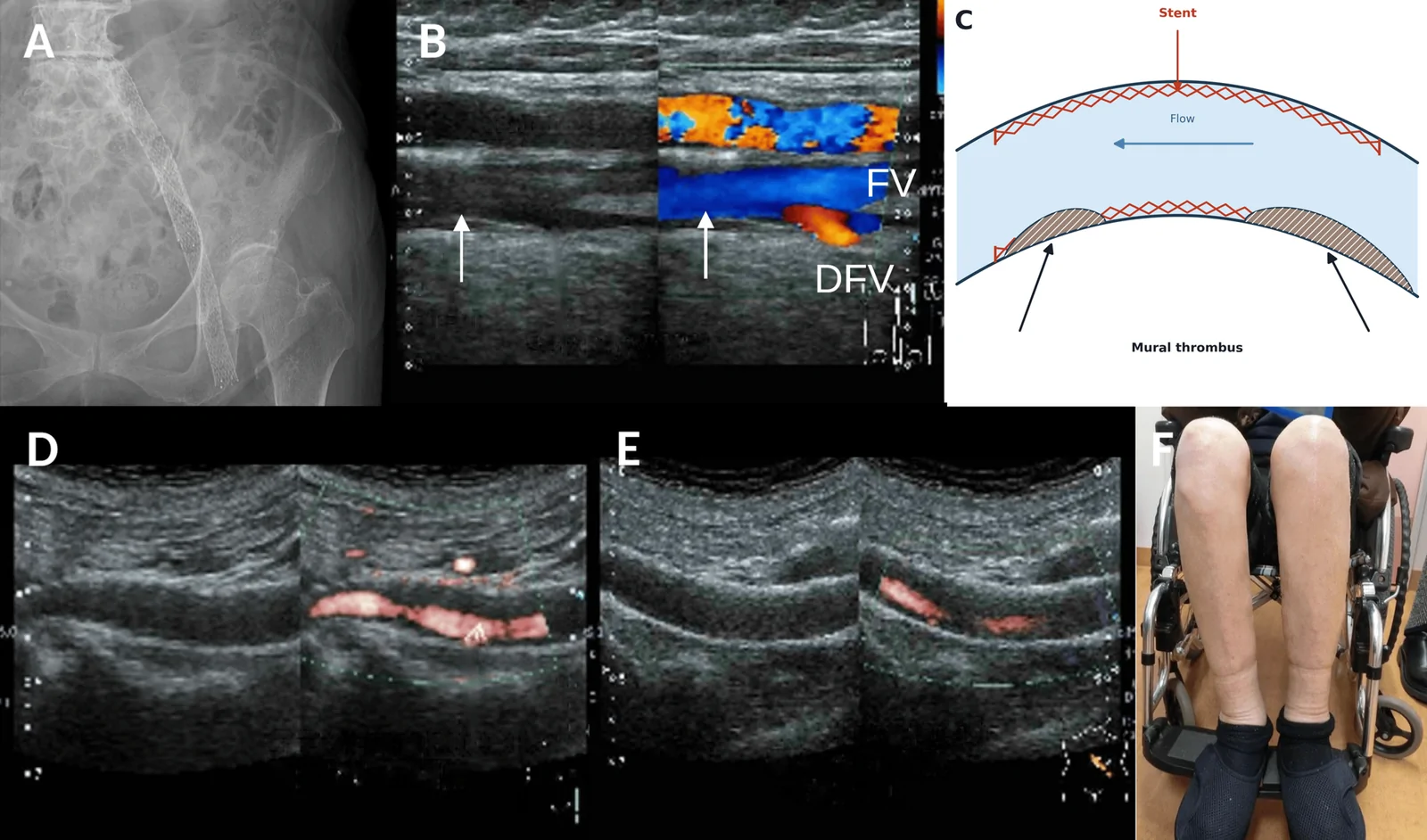
Postoperative imaging and serial duplex surveillance Immediate postoperative period: (A) Plain radiograph after deployment of two Venovo venous stents (Becton, Dickinson and Company (BD), Tempe, AZ, USA); the arrow indicates the deployed stent. (B) DUS (B-mode on the left, colour Doppler on the right) on postoperative day one; the arrows indicate the distal stent end in the common FV, 20 mm proximal to the femoral confluence, with preserved inflow from the FV and DFV. Surveillance: (C) Schematic of the DUS finding at three months; the stent is drawn in red, hatching marks the mural thrombus that formed along the posterior wall of the stent, the black arrows indicate its two components, and the blue arrow indicates the direction of venous flow. Image credits: Shinsuke Kikuchi. Panel C was prepared using Microsoft PowerPoint (Microsoft Corporation, Redmond, WA, USA). (D) DUS at six months; the arrow indicates the in-stent mural thrombus, measuring up to 5 mm. (E) DUS at 12 months; the arrow indicates the same mural thrombus, measuring up to 6 mm. Twelve-month status: (F) Photograph of the lower limbs at 12 months, showing no oedema. Abbreviations: DFV, deep femoral vein; DUS, duplex ultrasound; FV, femoral vein.

At one month, the clinician-assessed scores had improved, and DUS showed no reflux in the femoral or popliteal veins, less than 25% stenosis throughout the stented and native segments, and patent DFV and GSV. The patient herself, however, still rated her leg as considerably worse than one year earlier on the VEINES-QOL/Sym questionnaire and reported marked limitation of household and standing activities [7]. Compression stockings were used only intermittently at this point and were discontinued between one and three months, when her symptoms had resolved; as a 77-year-old woman unable to apply them unaided, she asked not to continue.

Three months after stenting, surveillance DUS detected a localised mural thrombus along the posterior wall of the stent, approximately 3 mm thick and extending over more than 2 cm in the longitudinal plane, with mild neointimal thickening (Figure 3C). Because the thrombus had formed despite edoxaban combined with aspirin, and because antiplatelet therapy could not be withdrawn given the arterial indication, aspirin was changed to clopidogrel 75 mg once daily. Colour Doppler showed preserved flow around the thrombus with maintained antegrade inflow from the FV and DFV; peak velocities within the stent were not recorded.

The thrombus nevertheless enlarged, measuring up to 5 mm at six months and 6 mm at nine and 12 months (Figures 3D, 3E). The 1 mm difference between the nine- and 12-month studies is within expected measurement variability, and the thrombus is best described as unchanged over the final six months rather than stable throughout. Mural thrombus and neointimal hyperplasia cannot be reliably distinguished on a single examination; our interpretation was based on the progressive increase in echogenicity across serial studies, which we took to indicate organising thrombus.

Antithrombotic therapy was interrupted three times during follow-up. At four months the patient reported rectal bleeding; edoxaban and clopidogrel were withheld for two days for colonoscopy, which identified haemorrhoids and no malignancy, and the bleeding was managed conservatively. At nine months she developed haematuria, and a bladder stone was identified; imaging performed during that work-up incidentally demonstrated a breast mass, and biopsy confirmed a triple-negative invasive breast carcinoma with nodal involvement. Her body weight had fallen from 60 kg at presentation to 54 kg. She underwent mastectomy at ten months, with three days of heparin bridging, and began adjuvant chemotherapy at eleven months. Transurethral lithotripsy was performed at 12 months with two days of heparin bridging.

At 12 months, the stent remained patent, the mural thrombus was unchanged, and radiography showed no stent fracture or migration. The limbs showed no oedema (Figure 3F).

Serial clinical outcome measures are summarised in Table 1. The rVCSS fell from 8 at baseline to 1 at 12 months, the Villalta score from 9 to 2, and the VDS from 2 to 0. CEAP clinical class improved from C3 to C0 by one month and remained so thereafter. Calf circumference in the treated limb was 31.5 cm at baseline and 30.7 cm at 12 months, while the contralateral limb measured 29.0 cm and 31.0 cm at the same time points; the side-to-side difference therefore fell from 2.5 cm to 0.3 cm.

**Table 1:** Serial clinical outcome measures Baseline measurements were obtained before stenting, three months after the acute deep vein thrombosis, and all baseline scores were recorded at the same visit. Time points are given as months after stenting. Calf circumference was measured 20 cm distal to the knee joint line. Scores were not recorded at nine months. CEAP, Clinical-Etiological-Anatomical-Pathophysiological classification; NPRS, numeric pain rating scale; rVCSS, revised Venous Clinical Severity Score; VDS, Venous Disability Score.

	Baseline	One month	Three months	Six months	12 months
CEAP clinical class [4]	C3	C0	C0	C0	C0
rVCSS [5]	8	4	1	0	1
Villalta score [6]	9	7	2	2	2
NPRS	3	0	0	2	0
VDS [5]	2	1	1	1	0
Calf circumference, treated limb (cm)	31.5	31.5	31.0	29.0	30.7
Calf circumference, contralateral limb (cm)	29.0	29.0	32.0	29.0	31.0

## Discussion

This case illustrates three points relevant to the early adoption of dedicated venous stenting in Japan.

First, IVUS influenced procedural decision-making in a way that venography did not. IVUS detects substantially more significant iliofemoral lesions than multiplanar venography [8]. Here, it identified a stenotic proximal CFV that was not apparent on venography, and this finding determined the decision to extend stent coverage distally. True-lumen measurements allowed device sizing to be matched to each segment, and the post-stenting lumen areas corresponded closely to the nominal stent diameters. The lesion was type 3 in the proposed anatomical classification, a category previously associated with favourable outcomes at the population level rather than one that guarantees patency in an individual patient [3]. Outcome data for these stents in Japanese practice remain sparse; in the pivotal VERNACULAR study, the Venovo stent achieved a 12-month primary patency of approximately 88%, with lower patency in post-thrombotic than in non-thrombotic lesions [9].

Second, and in our view the most instructive aspect of this case, a mural thrombus formed within the stent despite combined anticoagulant and antiplatelet therapy. The optimal antithrombotic regimen after venous stenting is not well established. Anticoagulation during the first six to 12 months is widely favoured, but there is no consensus on adjunctive antiplatelet therapy [10,11], and a recent international registry found that anticoagulation without an antiplatelet agent is the most common strategy [12]. In our patient, an antiplatelet agent was already indicated for peripheral artery disease, so the question was not whether to add one but which to use. When surveillance detected the thrombus, we changed aspirin to clopidogrel rather than withdrawing antiplatelet therapy. The thrombus nevertheless continued to enlarge for a further six months before reaching a plateau, and this sequence does not show that the change influenced the outcome; the same course might have been observed had aspirin been continued.

The subsequent diagnosis offers an alternative explanation. Nine months after stenting, the patient had lost 10% of her body weight, and an invasive breast carcinoma was diagnosed incidentally during investigation of a bladder stone. Occult cancer is detected in approximately one in 20 patients within 12 months of an unprovoked venous thromboembolism, whereas the figure after a provoked event is closer to 2% [13]. The tumour was triple-negative with nodal involvement, a subtype in which a more pronounced procoagulant profile has been described [14]. This thrombosis was classified as provoked on the basis of transient recumbency, and thrombophilia testing was therefore omitted; the later diagnosis raises the possibility that the event was not truly provoked and that an undiagnosed malignancy contributed from the outset. Whether the weight loss reflected the malignancy, the bladder stone, or both cannot be determined.

Third, compression therapy was discontinued between one and three months, when symptoms had resolved, at the patient's request; as a 77-year-old woman, she was unable to apply the stockings unaided. We accept that this was not ideal. Compression has been reported to reduce the incidence of post-thrombotic syndrome, and in retrospect, once a mural thrombus had been detected, resuming compression would have been a reasonable adjunct. The favourable outcome in this patient should not be read as evidence that compression can be omitted after venous stenting. It is nonetheless reassuring that the stent remained patent through two bleeding events and three interruptions of antithrombotic therapy, including heparin bridging for mastectomy and for lithotripsy.

Stents placed for post-thrombotic obstruction continue to fail beyond six months, in contrast to those placed for non-thrombotic lesions in which failure is largely confined to the first six months [15]. Given the post-thrombotic aetiology, the persistent mural thrombus, the intercurrent malignancy, and our limited institutional experience, we plan duplex surveillance every three months until two years, extending to six-monthly intervals thereafter if the thrombus remains unchanged, and intend to discontinue clopidogrel after the first year.

This report has limitations. It describes a single patient with 12 months of follow-up. No comparative evidence is available to show that the change from aspirin to clopidogrel caused the thrombus to stabilise or prevented its progression. Thrombophilia testing was not performed, so an underlying thrombophilic state cannot be excluded as a contributor. Peak velocities within the stent were not recorded, so the description of the thrombus as non-occlusive rests on preserved colour filling rather than on quantitative haemodynamic data. Calf circumference varied by up to 3 cm between visits in the untreated limb, indicating substantial measurement variability.

## Conclusions

A dedicated venous stent restored in-line flow and relieved symptoms in a limb with chronic post-thrombotic iliofemoral obstruction, and remained patent for one year. IVUS identified a stenotic distal landing zone that venography did not show.

A mural thrombus nevertheless formed within the stent despite anticoagulation combined with an antiplatelet agent, and preceded the incidental diagnosis of an invasive breast carcinoma by six months. This sequence raises the possibility that a thrombus arising within a venous stent despite adequate antithrombotic therapy may reflect an undiagnosed malignancy.
